# Preference for in-person psychotherapy versus digital psychotherapy options for depression: survey of adults in the U.S

**DOI:** 10.1038/s41746-019-0077-1

**Published:** 2019-02-11

**Authors:** Brenna N. Renn, Theresa J. Hoeft, Heather Sophia Lee, Amy M. Bauer, Patricia A. Areán

**Affiliations:** 1 Department of Psychiatry & Behavioral Sciences, University of Washington, Seattle, WA USA; 2 Department of Family Medicine & Community Health, Rutgers Robert Wood Johnson Medical School, New Brunswick, NJ Canada

**Keywords:** Human behaviour, Health services, Therapeutics

## Abstract

Several barriers complicate access to psychotherapy for depression, including time commitment, location of services, and stigma. Digital treatment has the potential to address these barriers, yet long term use of digital psychotherapy is poor. This paper presents data from a mixed-methods, online survey to document concerns patients with depression face when given the choice of in-person psychotherapy and digital psychotherapy. Participants were 164 adults living in the United States who had previously used or considered psychotherapy for depression. Rural-dwelling and racial/ethnic minority (Native American, African American, and Spanish-speaking) respondents were purposively sampled. Participants were asked their preferences for and opinions about four treatment modalities: self-guided digital, peer-supported digital, expert-guided digital, or in-person psychotherapy. Less than half (44.5%) of participants preferred in-person psychotherapy, 25.6% preferred self-guided digital treatment, 19.7% preferred expert-guided digital treatment, and 8.5% peer-supported digital treatment. Principal themes extracted from qualitative analysis centered on the efficacy of digital treatment, access to digital treatment, concerns about peer-supported care, confidentiality and privacy concerns, preference for in-person treatment, skepticism about self-guided therapy, and the impact of social anxiety on the use of video-chat based care. Future development of digital psychotherapy will need to address concerns regarding efficacy, privacy, data security, and methods to enhance motivation to use these treatments.

## Introduction

Depressive disorders are prevalent and are a leading cause of disability, lost productivity, and health care expenditure.^1^ Psychotherapies are effective in the treatment of mild to moderate depression,^2,3^ are first-line treatments for major depression,^4^ and are also the preferred treatment modality by most people with depression.^5–7^ Despite the effectiveness and preferences for these treatments, most people who seek psychotherapy for depression only attend one session,^8–10^ and in the US overall utilization by people with depression has declined from 15% in 1997 to 10% in 2007.^11–13^ At fault for poor utilization are transportation, time commitment (psychotherapy is typically delivered in weekly, hour-long appointments in a clinician’s office), and supply-side barriers, with too few professionals living in rural areas. The stigma that many cultural groups have related to seeking care also is noted as an important utilization barrier.^14–17^ A potential solution to overcome these barriers is providing treatment via digital means, such as on-line tools and mobile applications (apps). Currently, digital psychotherapy consists of self-guided digital treatment, in which consumers use web-based and app-based care to apply therapeutic principles without any additional support; peer-guided digital treatment, in which consumers use digital devices with access to trained peer support specialists who support and guide their use of the treatment program; and expert-guided digital treatment, in which consumers use digital devices with help from a professional. Studies find that digital interventions are efficacious,^18^ particularly for guided digital treatment,^19^ although effect sizes are smaller than those found for face-to-face treatment,^20^ and have negligible cost savings.^21^ Healthcare organizations around the globe offer digital tools to their depressed patients and a new healthcare industry based on fee-for-service, subscription based digital treatment has emerged in the US.^22^

While many digital interventions have not been rigorously tested, some digital interventions are evidence-based,^23–25^ and may address access barriers, a growing evidence base finds that utilization of digital treatment mirrors that of in-person psychotherapy and many patients with depression may prefer in-person, face-to-face treatment.^26,27^ For example, one study found that 86% of patients surveyed preferred face-to-face care to on-line treatment^27^ and another found that those who are reluctant to use in-person treatment are also reluctant to use on-line treatment.^28^ Although most people indicate a willingness to try digital interventions, there is some indication that concerns about effectiveness may mitigate use.^29^ Integration of digital treatment into health care systems is no small investment^21^ and more research is needed to ascertain why depressed patients maintain a preference for in-person psychotherapy, compared to digital treatment options.^30^ The purpose of this study is document concerns people face when offered a choice between self-guided digital programs, digital programs with coaching options, or traditional in-person psychotherapy.

## Results

### Participant characteristics

See Table 1 for sample characteristics. Of the 238 individuals who responded to the survey, 56 (23.5%) were ineligible, and 18 (7.6%) did not complete the survey. The analytic sample was comprised of 164 eligible participants between 21–70 years of age (*M* = 32.88, SD = 8.29), 49.4% of whom identified as racial or ethnic minority, and 10.4% were Spanish speakers. Our respondents were equally distributed across urban (51.8%) and rural (48.2%) locales in the U.S. Approximately half (*n* = 81, 49.4%) of the respondents had previously received individual in-person psychotherapy from a licensed clinician, and 17.1% (*n* = 28) had used a mobile app for self-help or self-monitoring of depressive symptoms. Owing to a survey error, we were not able to accurately determine gender distribution; the survey program failed to populate this variable.

**Table 1:** Participant characteristics

	Mean (SD) or *n* (%)
Age	32.88 (8.29) range 21–70 years
Race	
African American/Black	34 (20.7)
Asian/Asian American	6 (3.7)
American Indian/Alaska Native	24 (14.6)
Native Hawaiian/Pacific Islander	0 (0)
White	91 (55.5)
More than one race	7 (4.3)
Unknown	2 (1.2)
Hispanic (*n*, % yes)	23 (14.0)
Rural-dwelling (*n*, % yes)	79 (48.2)
Previous treatment for depression (*n*, % yes)	
Psychotherapy with licensed clinician	81 (49.4)
App for self-help or self-monitoring	28 (17.1)
Either previous treatment (psychotherapy or self-help app)	87 (53.0)

### Access barriers

Participants were asked to select barriers they had previously encountered when considering in-person psychotherapy; the top three barriers endorsed by this sample were cost (48.2%), insurance limitations (26.8%), and stigma (26.2%). There were no demographic differences in the endorsement of barriers.

### Preferences for in-person versus digital treatment

Seventy three percent of the sample would likely try individual in-person psychotherapy and 72% would try digital psychotherapy in the future. When forced to choose between modalities, the majority (44.5%) preferred individual in-person psychotherapy. Only a quarter (25.6%) of respondents preferred self-guided digital treatment. The options of self-guided digital psychotherapy enhanced with peer or expert support were least preferred (8.5% and 19.7% of the sample, respectively). Although 17.7% of respondents endorsed concerns with individual in-person psychotherapy, more than a quarter (27.4%) of the sample endorsed concerns about self-guided digital treatment, 36.8% of the sample endorsed concerns about digital treatment supplemented with peer support, and 36.8% expressed concerns regarding digital treatment supplemented with expert support. See Table 2.

**Table 2:** Preferences for and concerns regarding treatment modality

	Frequency *n* (%)		
	Yes	No	Unsure
*Would consider in future*			
Individual in-person	120 (73.2)	20 (12.2)	22 (13.4)
Self-guided online	118 (72.0)	23 (14.0)	21 (12.8)
Self-guided plus peer support*	64 (54.7)	28 (23.9)	25 (21.4)
Self-guided + peer + provider chat*	66 (56.4)	26 (22.2)	25 (21.4)
*Most likely to choose in future*			
Individual in-person	73 (44.5)	–	–
Self-guided online	42 (25.6)	–	–
Self-guided plus peer support*	10 (8.5)	–	–
Self-guided + peer + provider chat*	23 (19.7)	–	–
*Endorsed concerns*			
Individual in-person	29 (17.7)	133 (81.1)	–
Self-guided online	45 (27.4)	117 (71.3)	–
Self-guided plus peer support*	43 (36.8)	74 (63.2)	–
Self-guided + peer + provider chat*	43 (36.8)	74 (63.2)	–

All other calculations are based on the entire sample (*N* = 164).

*A subset of the sample (*n* = 117) received additional questions specifically about peer support supplements to self-guided online treatment

Preference for digital versus in-person psychotherapy did not vary by rural/urban status, racial/ethnic minority status, or by age. However, 91.4% of those who had previously had in-person psychotherapy with a licensed clinician were more likely to re-consider this treatment in the future, compared to 64.4% of those without previous in-person psychotherapy experience (*t*(160) = −3.56, *p* ≤ 0.001).

### Concerns regarding treatment selection

In the qualitative data, the following themes emerged from the open-ended responses: (1) concerns about relative effectiveness for digital treatment compared to in-person treatment; (2) access barriers; (3) skepticism specific to self-guided therapy; (4) social anxiety; (5) preference for in-person treatment (6) concerns about confidentiality or privacy; and (7) concerns specific to peer support. Table 3 illustrates themes and subthemes, along with the percentages of sample endorsing these codes. Subthemes were identified by at least three of the 164 respondents.

**Table 3:** Preferences for and concerns regarding treatment modality

Theme and subthemes	Percent of sample endorsed (*n*)
Theme 1: relative effectiveness questions and concerns	62.8% (103)
Questions about relative treatment outcome	50.6% (83)
Adherence and self-efficacy to complete self-guided treatment	22.0% (36)
Lack of professional monitoring	4.3% (7)
Theme 2: access barriers	31.7% (52)
Theme 3: skepticism of self-guided therapy	12.8% (21)
Not personalized enough	4.3% (7)
Concerns about safety of self-guided	6.1% (10)
Skepticism or mistrust about app developers	2.4% (4)
Theme 4: social anxiety	12.8% (21)
Unease with video chat	7.3% (12)
Unease with in-person therapy	6.7% (11)
Theme 5: preference for in-person treatment	13.4% (22)
Theme 6: concerns of confidentiality or privacy	15.2% (25)
Concern with lack of confidentiality/privacy for in-person therapy	3.0% (5)
Concern with lack of confidentiality/privacy for online therapy	12.2% (20)
Theme 7: concerns with peer support	16.2% (19)*
Skepticism that peers are effective or well-trained	13.7% (16)*
Lack of trust or comfort with peers	4.3% (5)*

All other calculations are based on the entire sample (*N* = 164).

*A subset of the sample (*n* = 117) received supplemental options about peer-support

#### Theme 1: relative effectiveness

Sixty two percent of the participants raised questions or concerns regarding the relative efficacy or effectiveness of digital psychotherapy and in-person psychotherapy. Over half the sample (50.6%) raised questions about relative success rates between in-person psychotherapy versus digital psychotherapy (Box 1, data extracts 1–2). Twenty two percent of the respondents questioned the effectiveness of self-guided digital psychotherapy. A primary concern raised here was lack of accountability to a therapist (Box 1, data extract 3). Some respondents specifically indicated that having personal contact was important to address motivational issues related to depression (Box 1, data extracts 4–5). A subset of participants (4.3%) also shared unease about the perceived lack of professional monitoring in self-guided digital psychotherapy and its impact that has on treatment efficacy and effectiveness (Box 1, data extracts 6–7).

#### Theme 2: access barriers

Almost one-third of the sample (31.7%) remarked that access barriers would factor into their decision regarding choice of treatment. Most notable was the issue of cost (Box 2, extract 1). Participants reconfirmed that known in-person access barriers remained (e.g.: scheduling conflicts, and competing demands (e.g., family responsibilities (Box 2, extract 2). Practical concerns related to digital treatment were whether health insurance would cover this care, what equipment would be needed, frequency of sessions, and duration of treatment.

#### Theme 3: concerns with peer support

Sixteen percent of the sample had negative comments about peer support. Thirteen percent reported skepticism that peers are effective or well-trained, 4.3% reported discomfort related to trust, professionalism, and general discomfort (Box 7, extracts 2–5).

#### Theme 4: concerns about confidentiality and privacy

Privacy and confidentiality were notable concerns for 15.2% of respondents. These responses typically applied to concerns with digital psychotherapy (12.2%), although a subset of consumers (3.0%) commented on privacy or confidentiality concerns seeking in-person psychotherapy. Some of these issues were rooted in fear of stigma and a lack of privacy using shared technology at home (Box 6, data extract 1). Others specifically noted concerns about information security in the context of digital treatment, including fears of data breaches or unwanted recording or sharing of their sensitive information (Box 6, data extracts 3-4).

#### Theme 5: preference for in-person psychotherapy

Thirteen percent of participants articulated a preference for in-person psychotherapy, specifically their beliefs in this treatments’ superior outcomes and that it was more authentic and private (Box 5, extract 1). For others, a history of in-person psychotherapy informed their preference for that modality (Box 5, extract 3).

#### Theme 6: skepticism of self-guided digital psychotherapy

Nearly 13% of respondents were skeptical of self-guided digital treatment. Approximately 4% of respondents felt self-guided treatment was not personalized (Box 3, extracts 1–2). Six percent raised concerns over the safety of self-guided digital psychotherapy (Box 3, extracts 3–4). A small percent (2.4%) raised concerns about the trustworthiness of the intervention developer and questioned their motives (e.g., profit; Box 3, extract 5).

#### Theme 7: social anxiety

Nearly thirteen percent of respondents stated that their social anxiety would interfere with either the use of in-person therapy or a video chat element (such as that used to supplement digital treatment with peer or expert support; 7.3%). Those who expressed unease with video chat commented on the artificiality and discomfort associated with this technology (Box 4, extract 1). Four of these respondents specifically highlighted “social anxiety” as concerns with video-chat (Box 4, extracts 2-3). These participants shared they were uncomfortable or even ashamed about disclosing their “problems” or feelings, particularly to a stranger (Box 4, extracts 4-6). However, some might be willing to overcome this barrier if they perceived a need for treatment (Box 4, extract 7).

Box 1 Theme 1, relative effectiveness: extracts from responses

1. 32 y/o urban Asian American respondent: “What are the success rates? How do the two [in-person versus self-guided] compare?”
2. 44 y/o urban non-Hispanic White respondent: “Have there been any studies to determine if self-guided therapy actually works?”
3. 36 y/o rural African American respondent: “I would be worried that I wouldn’t be as motivated to complete the sessions if there was no appointment and nobody to hold me accountable.”
4. 35 y/o rural African American respondent: “I am not self-motivated. I need an actual human to push me in the right direction. I suffer from depression—I need to talk to a human.”
5. 31 y/o urban non-Hispanic White respondent: “With depression comes a lack of motivation—how will this affect self-guided therapy?”
6. 30 y/o urban Hispanic respondent: “How would I know I am doing well by myself? What if it isn’t enough? How would I even know?”
7. 32 y/o rural non-Hispanic White respondent: “I wouldn’t be sure if I was coming to the correct conclusion about my mental health. I would like to have professional guidance.”

Box 2 Theme 2, access barriers: extracts from responses

1. 27 y/o rural mixed-race respondent: “I would ask about how much each type of therapy would cost.”
2. 29 y/o urban mixed race/Hispanic respondent: “How long does [treatment] last? Will it be weeks or months?”
3. 46 y/o rural non-Hispanic White respondent: “How flexible would the one-on-one scheduling be? Would I need to make big chunks of time available? Or commute?”
4. 34 y/o urban non-Hispanic White respondent: “I’d want to know how much time each [type of treatment] would take. I’d want to know the cost of them and if my insurance covered any or all of it.”
5. 27 y/o rural Native American respondent: “I like the idea of therapy without having to go to another town.”

Box 3 Theme 3, concerns with peers: extracts from responses

44 y/o urban non-Hispanic White respondent: “I feel hesitant to accept help from trained peers. Who are these peers? How are they trained? Would they know who I was? Are they bound by the same confidentiality requirements as actual therapists? I don’t see trained peers getting the same type of education and training as a licensed clinician.”
31 y/o urban non-Hispanic White respondent: “My biggest concern would be about my peers. I’m not sure I would feel comfortable interacting with other people who aren’t exactly professionals.”
25 y/o urban non-Hispanic White respondent: “I’m just not sure how much I would trust trained peers if they weren’t licensed.”
30 y/o urban mixed race respondent: “I’m concerned about ‘trained peers’ and how they would treat my medical information.”
5) 30 y/o urban non-Hispanic White respondent: “I would be concerned the peer feedback wouldn’t be tailored or professional enough for my needs.”

Box 4 Theme 4, concerns about confidentiality and privacy: extracts from responses

27 y/o rural Native American respondent: “I would be a bit self-conscious [using home video conferencing] about anyone walking in and seeing me at home.”
21 y/o rural Native American respondent: “People I know might see me seeking [in-person] help.”
42 y/o urban non-Hispanic White respondent: “I am not sure I would want to do the video[chat] for fears it could be recorded or spied on by others without my consent.”
4) 30 y/o rural African American respondent: “I would have privacy concerns. I would be worried that the information that was talked about through text or video could be recorded and accessed by unauthorized people.”

Box 5 Theme 5, preference for in-person treatment: extracts from responses

1. 38 y/o urban non-Hispanic White respondent: “I would do the in-person therapy. I would need to be able to get feedback and be held accountable. If I did it online, I could lie and not really get any help. I would get the help I need in the room with a counselor.”
2. 46 y/o urban non-Hispanic White respondent: “One of the benefits of therapy for me was the actual human interaction. My depression was bad enough that I isolated myself. The very act of getting ready to leave the house, dressing, showering, putting on makeup, getting in the car and going somewhere - were extremely helpful. Then to be there and sit with the doctor and look another human being in the face/eye, feel a human connection with him, have him express sympathy or laugh with me—so important. How do you get that online or in an app? I think you need to make sure they are a part of therapy somehow.”
3. 38 y/o rural non-Hispanic White respondent: “I like the in-person [option]. It made a big difference to me.”

Box 6 Theme 6, skepticism: extracts from responses

1. 60 y/o rural non-Hispanic White respondent: “I would feel like [self-guided therapy] is very ‘cookie-cutter’-ish. I would not feel the insights were personal but more textbook recommendations. If it were the absolute only option, then I might consider online therapy but at that point I might as well just read a book.”
2. 25 y/o urban non-Hispanic White respondent: “Self-guided therapy may not be helpful because I very much need someone to teach me how to cope with depression and anxiety. I feel like I wouldn’t learn much [in self-guided treatment].”
3. 27 y/o rural Native American respondent: “I’d be concerned about doing it [self-guided therapy] right.”
4. 28 y/o rural Native American respondent: “I worry that it [self-guided therapy] would bring up emotions that I would not be able to manage alone.”
5. 53 y/o urban Asian American respondent: “I would ask [my doctor recommending self-guided treatment] who developed the online-based program and were there any studies comparing different types of therapy? I would also ask if the program had been analyzed by any industry associates.”

Box 7 Theme 7, social anxiety: extracts from responses

1. 32 y/o urban non-Hispanic White respondent: “My concern would be the video chat itself. I’d be worried that my responses and behavior would be different due to being on camera. I wouldn’t feel [like] myself and would probably speak less about my issues because of that insecurity I’d be feeling.”
2. 42 y/o urban non-Hispanic White respondent: “I have severe social anxiety, so video conferencing would be out of my comfort zone.”
3. 43 y/o urban non-Hispanic White respondent: “Since I suffer from social anxiety I think I would have some trouble getting in front of a camera. This obstacle is really not that different from seeing a therapist in person.”
4. 28 y/o rural African American respondent: “I will feel bad discussing my depression in person as I am concerned what other people will think of me.”
5. 30 y/o rural Native American respondent: “I am too withdrawn to talk to someone about how I feel. I am ashamed of how I feel and have a hard time talking about my feelings.”
6. 27 y/o rural Hispanic respondent: “I am afraid to completely open up [in in-person therapy].”
7. 29 y/o urban non-Hispanic White respondent: “It makes me anxious, nervous, and uncomfortable to consider seeing a professional in person, but if I did that it would be because I thought it was necessary.”

## Discussion

Several key findings were identified in this mixed methods survey of U.S. adults. First, much of the sample indicated a preference for in-person psychotherapy, even though they acknowledged that known access barriers such as transportation, cost, and stigma remain.^11,16^ The preference for in-person psychotherapy appears to be driven by concerns about the relative efficacy of digital psychotherapy compared to in-person psychotherapy, concerns regarding privacy and data security and whether digital psychotherapy is covered by insurance. These findings have been identified in other studies.^27–29,31–33^ Privacy and data security concerns are likely due to recent reports of digital breaches by large technology companies.^34,35^ Concerns about relative effciacy of digital psychotherapy compared to in-person psychotherapy could be addressed through broader dissemination of researching finding positive effects of digital psychotherapy, as well as the potential advantages of digital psychotherapy over in-person psychotherapy (e.g.: immediate access to a provider). Privacy concerns are harder to address, particularly given that some concerns regarding privacy had to do with limited access to private computers for care. Data security is also harder to address; as of yet there are no uniform standards for protection of information collected from digital therapeutics. The need to assure digital privacy is echoed by the guidelines for mental health apps developed by the American Psychiatric Association.^36^ They recommend that developers prioritize privacy and security. In sum, the concerns regarding digital psychotherapy (relative efficacy, patient protection, and personalization) are significant enough impact potential utilization of these treatments and will need to be addressed if there is to be routine use of digital psychotherapy.^37^

Other key findings from this survey were the concerns raised about specific types of digital psychotherapy. In conducting this survey, we were particularly interested in acceptability of a stepped care model of digital psychotherapy, where people could opt into different levels of care, namely, self-guided, peer-guided, and expert-guided digital psychotherapy. Strikingly, self-guided digital psychotherapy was the more preferred model of digital care, with a quarter of the sample saying they would opt for this treatment. However, many concerns were raised about the effectiveness of this model of care, in particular the lack of personalization, accountability, and potential safety risks of not having a provider monitor outcomes. These issues potentially explain why several studies have found poor uptake and continued use of self-guided care, particularly when it is compared to expert-guided care.^38^

It is possible that preference for self-guided care was influenced by the way we described this treatment to participants, that treatment would be delivered via video-chat and included an optional peer support component. Several participants had concerns regarding the qualifications of peer counselors, and many participants indicated that video-chat was not a technology they felt comfortable using. Although peer-supported care has been adopted in certain populations and settings, such as among veterans, those with serious mental illness, and individuals with chemical dependence, peer support is typically built into mental health treatment teams as an adjunct to traditional care.^40^ The reactions our participants had to peer support may be based on their experience with social media, in which individuals received unregulated advice from untrained peers in unregulated chat rooms or websites. Respondents may have confused our questions about “support from trained peers” with those encounters in this unregulated space. Our findings imply that providers or treatment developers who incorporate peer support services into care should make clear the qualifications and role of peer providers in providing support services, as well as any data on the effectiveness of peer-supported treatment.

To our knowledge, this is the first study to use a mixed-methods approach to ascertaining concerns faced by patients with depression. Further, using a bilingual online survey modality allowed us to overcome access barriers and oversample those most underrepresented in mental health services and research, including Spanish speakers, Native Americans, African Americans, and individuals residing in rural areas. However, a number of limitations are highlighted here. First, our survey was conducted using an on-line survey tool, and thus this is a sample of people who are familiarity with on-line technology. Preferences and concerns raised by this sample may not generalize to the broader population who are not regular users of technology. A second limitation is our inability to conduct analysis based on gender. A recent study found that women are more likely to engage in on-line treatment than men, suggesting that gender remains another barrier in the use of psychotherapy.^28,41^ Unfortunately, we are not able to discern if any of the concerns and questions raised here were specific to gender groups. Third, although half of the respondents reported past experience with in-person psychotherapy, very few had previously tried online therapy or mental health apps. Prior exposure to psychotherapy may have influenced these results.^6^ Finally, our wording of digital psychotherapy in the survey referred to this class of treatment as “on-line psychotherapy.” Although we had explained the intervention included internet and app-based care, we did not explicitly ask participants to compare preferences based on type of digital psychotherapy.

In sum, these data provide useful information regarding the use of digital psychotherapy. Concerns raised by respondents can be addressed through more rigorous research and dissemination of the efficacy of digital treatments and through better management of data security and privacy. We suggest transparency and proactive attempts to address potential concerns about treatment effectiveness, privacy and confidentiality concerns, and the specific training and professionalism of any peers used to supplement treatment. In clinical settings, this is best accomplished using shared decision making, a collaborative process whereby clinicians present patients with information regarding the medical condition and its treatment options, and patients inform clinicians about their values, goals, experiences, and preferences regarding treatment. This patient-centered approach can lead to treatment and delivery refinements and better harness the potential of digital psychotherapy to transform the delivery of care and close treatment gaps.

## Methods

### Participants

Participants were adults (age ≥18 years) living in the United States who had previously considered either psychotherapy or the use of self-guided treatment (e.g., a mobile app or online self-help platform) for depression. These participants were preregistered in the Amazon.com, Inc. Mechanical Turk (MTurk) participant pool, described below, and self-selected to complete our survey examining consumer opinions of online/app-based versus in-person psychotherapy. This study was approved by the IRB at University of Washington. Participants were provided written information about the study and were required to complete an informed consent to participate.

### Procedures

Recruitment for the cross-sectional survey, described below, was conducted over four separate days between November 2016 and April 2017. Recruitment occurred in two rounds; minor modifications were made to the first survey based on initial results, stakeholder guidance, and our intention to purposively sample underrepresented groups with poor access to psychotherapy, in particular, we purposively recruited individuals who self-identified as Spanish speakers, Native American, African American, and rural dwellers. The survey was administered through MTurk. MTurk is an online crowdsourcing marketplace owned by Amazon that allows users to take surveys and perform other computer-based tasks posted to the marketplace. Previous research has found that crowdsourcing platforms like MTurk allow for rapid and inexpensive capture of high-quality survey data from a large and potentially more diverse population than typically seen in standard convenience samples, allowing for speed of innovation.^42^ The surveys were open to each registered MTurk user, who volunteered to participate and qualified based on endorsement that the user had previously considered psychotherapy for depression. The surveys were offered in both English and Spanish. Participants were told that “researchers at the University of Washington were interested in consumer opinions about in-person and online psychotherapy/counseling.” Amazon worker identification numbers are collected for payment; these numbers are unique to each user and ensured there were no duplicate responses. Survey responses were collected using Research Electronic Data Capture (REDCap)^43^ hosted at the Institute of Translational Health Sciences. REDCap (Research Electronic Data Capture) is a secure, web-based application designed to support data capture for research studies, providing: (1) an intuitive interface for validated data entry; (2) audit trails for tracking data manipulation and export procedures; (3) automated export procedures for seamless data downloads to common statistical packages; and (4) procedures for importing data from external sources. No personal data were collected or stored. Participants were paid $5 for completion. Completion checks were built into RedCap and response completeness was also checked by the first author (B.N.R.) after questionnaire submission.

### Measures

Survey questions were developed by the principal investigators and an advisory board consisting of representatives from U.S. national health plans (providers and administrators) and national patient advocacy groups. The survey was created to inform research questions for future comparative effectiveness research of digital treatment. The first survey focused specifically on preferences between in-person versus digital treatment (including on-line and app-based, self-guided and expert-guided, to include secure videoconferencing with a licensed clinician). The second survey had overlapping questions with the original survey and asked additional questions about the use of trained peer counselors to supplement digital treatment. The survey was comprised of structured questions and free text for qualitative analysis (see supplementary appendix). Participants were asked to rank their preferences for in-person versus different types of digital treatment and endorse any concerns with these treatment modalities. Qualitative analyses focused on the following open-ended questions:

- What concerns would you have about the following treatment options (individual in-person therapy, self-guided online therapy, and online therapy supplemented with support and/or videoconferencing with a licensed clinician)?
- If your doctor offered you the choice of getting therapy using an online-based program that offered self-guided therapy OR in-person therapy, what questions would you ask your doctor about the two types of treatment?
- Do you wish to share any additional comments about in-person versus online therapy/counseling?

For a subset of respondents (*n* = 117), the response options to questions #1 and 3 above were expanded to include peer support supplements to online treatment.

Because of small sample sizes across minority groups, racial and ethnic identity were collapsed to create a binary variable of non-Hispanic White and racial/ethnic minority status (encompassing Hispanic White, as well as Hispanic and non-Hispanic individuals of African/African American, Asian/Asian American, Native American/Alaska Native, Native Hawaiian/Pacific Islander, and multiracial identities).

### Data analysis

Data were analyzed using a complementarity mixed methods approach.^44^ Combined qualitative and quantitative methods have broad appeal in health research due to the ability of the two approaches to inform one another; in particular, inductive qualitative studies may provide the “why” to questions of intervention effectiveness and explore consumer perceptions of barriers and facilitators of treatment.

#### Quantitative data analysis

We calculated means and frequencies to report the sample demographics. Access barriers to in-person psychotherapy, preferences for treatment type, and concerns about treatment modalities were calculated as a percentage of the sample indicating such responses. Independent samples *t*-tests compared differences between demographic groups on stated preferences for psychotherapy modalities. All quantitative analyses were conducted in IBM SPSS Statistics, version 19.^45^

#### Qualitative data analysis

We imported participant responses to open-ended questions into Microsoft Excel. Two coders (P.A.A. and B.N.R.) sorted responses and developed themes using a grounded theory approach, whereby themes were derived inductively through open coding of the survey, rather than guided a priori by a theory or the literature.^46^ The themes were independently arrived at by the first two coders, and then verified by a third coder (H.S.L.), particularly in instances when the first two coders were not able to classify a response or when there was disagreement. Data were iteratively reviewed (open coded),^47^ and collapsed to mutually exclusive themes (axial coding). Once themes emerged in this way, the respondents’ comments were allocated to themes and the number of respondent comments that fell under a theme was tallied. On occasion, participant comments would reflect two themes. In those cases, (*N* = 5), the responses were counted in both categories. Given that there were no differences on preferences by demographic characteristics, qualitative themes are presented in the aggregate across participants.

## Data Availability

The data generated during an/or analyzed in this study are available from bnrenn@uw.edu on reasonable request or may be accessed via Synapse, and open access data repository, https://www.synapse.org/#!Synapse:syn17114082/files/.
